# Electrospun non-wovens potential wound dressing material based on polyacrylonitrile/chicken feathers keratin nanofiber

**DOI:** 10.1038/s41598-022-19390-3

**Published:** 2022-09-14

**Authors:** Eman Serag, Asmaa M. Abd El-Aziz, Azza El-Maghraby, Nahla A. Taha

**Affiliations:** 1 Marine Pollution Department, Environmental Division, National Institute of Oceanography and Fisheries, Kayet Bey, Elanfoushy, Alexandria, Egypt; 2 Fabrication Technology Research Department, Advanced Technology and New Materials Research Institute (ATNMRI), City of Scientific Research and Technological Applications (SRTA-City), Alexandria, Egypt; 3 Modeling and Simulation Research Department, Advanced Technology and New Materials Research Institute (ATNMRI), City of Scientific Research and Technological Applications (SRTA-City), Alexandria, Egypt

**Keywords:** Biotechnology, Materials science, Nanoscience and technology

## Abstract

Electrospinning nanofibers have a tremendous interest in biomedical applications such as tissue engineering, drug administration, and wound healing because of their ability to replicate and restore the function of the natural extracellular matrix found in tissues. The study’s highlight is the electrospinning preparation and characterization of polyacrylonitrile with chicken feather keratin as an additive. In this study, keratin was extracted from chicken feather waste using an environmentally friendly method and used to reinforce polymeric nanofiber mats. Scanning electron microscopy, energy dispersive spectroscopy, and transmission electron microscopy were used to examine the morphology and the structure of the prepared nanofiber mats. The effect of keratin on the porosity and the tensile strength of reinforcing nanofibers is investigated. The porosity ratio of the nanofiber mats goes up from 24.52 ± 2.12 for blank polyacrylonitrile (PAN (NF)) to 90.89 ± 1.91% for polyacrylonitrile nanofiber with 0.05 wt% keratin (PAN/0.05% K). Furthermore, keratin reinforcement improves the nanofiber's mechanical properties, which are important for wound dressing application, as well as its antibacterial activity without causing hemolysis (less than 2%). The best antibacterial activities were observed against *Pseudomonas aeruginosa* (30 ± 0.17 mm inhibition zone) and *Staphylococcus aureus* (29 ± 0.31 mm inhibition zone) for PAN/0.05% K sample, according to the antibacterial test. This research has a good potential to broaden the use of feather keratin-based nanofibers in wound healing.

## Introduction

The human skin acts as a large physical barrier, protecting our internal organs from mechanical damage, microbial infection, extreme heat, and ultraviolet radiation^1^. The skin, as the body's external epithelium, maintains organismal homeostasis and heals injuries throughout one's lifetime. So it is critical to protect the skin, which is the largest organ in the body^2^. Wounds can happen in everyday life. It imposes a monetary cost on society in proportion to the severity of individual suffering^3^. According to Leigh, the cost of work-related injuries and diseases in the United States is around $250 billion, or 1.8 percent of GDP. The Health and Safety Executive (HSE) estimated that work-related injuries and diseases cost the UK £14 billion, or about 1% of GDP. According to Safe Work^4^, workplace injuries and diseases cost Australia $61 billion, or 4.8 percent of GDP. Using the proper dressing increases cell proliferation, decreases healing time, and improves the individual quality of life^5^.

Nanotechnology is a straightforward method for producing nanofibers and nonwovens utilizing a high-voltage electrostatic field^6,7^. Electrospun non-woven wound materials have been extensively studied. Because they can be designed to prevent bacterial invasion, promote cell proliferation, as well as adsorption of wound exudates and dehydration of the wound surface^8,9^. Furthermore, because of their microporous nature, they can act as the extracellular matrix (ECM) by promoting cell adhesion and migration^10,11^.

Nanofibers for wound dressing come in a variety of shapes and sizes, they can be prepared from a variety of polymers (natural or synthetic), or they are made by blinding more than one type. Furthermore, biological molecules can be incorporated into the polymer matrix^12^. Because of its outstanding properties such as chemical stability, light resistance, non-toxicity, electrospinning, and mechanical elasticity, polyacrylonitrile (PAN) is a popular polymer for wound dressing^13^.

PAN, or its polymer composites, have been used in medical applications in addition to the different bio-derived compounds or nanoparticles. Many researchers have successfully created nanofiber composites by incorporating additional bioactive nanoparticles such as AgCl^14^, TiO_2_^15^, and silica^16,17^.

Keratin is a sort of natural physical protein found in profusion in hair, feathers, and wool, about 7–20% of cysteine is found in keratin^18^. Keratin-based materials have been demonstrated to have tremendous potential for usage in the biomedical area, especially in hemostasis^19^ and wound healing^20^. Previous research has found that keratins not only aid in wound healing by accelerating hemostasis and promoting cell growth but also increase the expression of several proteins that are important. Keratin, on the other hand, can help to promote increasing hemostatic speed by hastening plasma coagulation and increasing the lateral growth of fibrils. On the other hand, keratin's ability to promote attachment, proliferation, and produce collagen of fibroblasts, may also aid wound healing^21^. Non-toxic wound dressings that can increase cell proliferation are ideal. Wound dressings have included chitosan, gelatin, and other natural ingredients. Keratin has grown in prominence as a result of its high biocompatibility and biodegradability^22^. Keratin's unique peptide sequences Leu–Asp–Val, and Leu–Asp–Val can improve cell growth, collagen deposition, fibroblast adhesion, and keratinocyte migration^23^. According to Park et al.^24^, human hair keratin-based hydrogels were more effective at wound healing than wool keratin-based hydrogels. Tan et al.^25^ developed a porous keratin/chitosan scaffold with excellent antibacterial and cell proliferation properties. Moreover, Wang's team has created porous feather keratin films for controlled drug delivery. Due to its low cost and ease of processing, keratin may be considered a viable alternative to collagen for wound dressing materials.

We prepared an innovated PAN/ K nanofiber mats for wound healing as a potential application. A PAN nanofiber containing keratin as a bioactive membrane was created for wounds such as burns and diabetes-related ulcers. Chicken feathers were used to extract keratin, which was added with PAN and spun into fibers using the electrospinning techniques. Finally, we examined the properties of these mats, including porosity ratio, tensile strength, microstructures, hemocompatability, cytoxicity, and antibacterial activity.

## Materials and methods

### Materials

Chicken feathers were gotten from the local market. Polyacrylonitrile (PAN) with MWT of 150,000 g/mol was purchased from Sigma Aldrich (USA). N,N-Dimethyl Formamide (C_3_H_7_NO) 99.0%, sodium hydroxide (NaOH), formaldehyde (36%), calcium chloride, PBS buffer (pH 7), and acetic acid (CH_3_COOH) were purchased from El-Goumhouria Company, Alexandria, Egypt. Acid citrate dextrose solution (ACD) was purchased from Sigma-Aldrich (Chemie GmbH, Steinheim, Germany). Gram-negative *Pseudomonas aeruginosa ATCC 27853* (*P. aeruginosa*) and gram-positive *Staphylococcus aureus ATCC 25923 (S. aureus*) bacteria were investigated for antibacterial tests. For biomedical tests (hemolysis and thromogenicty tests), the blood samples were obtained from rabbits.

### Methods

#### Isolation of Keratin from chicken feather

The isolation of keratin from chicken feathers according to^26,27^ involves collecting, washing, drying, and grinding the feathers. The 5% w/v blended feather was dissolved in sodium hydroxide (1N) at 70 °C for 3 h. Acetic acid was added until the pH reached 7, then the hydrosolated feathers were transferred to a water bath to form a gel. The formulated gel was dried at 70 °C until it was fully dry. The extraction rate of feathers was calculated as the difference in weight between the chicken feather and the extracted keratin divided by the initial weight of feather as the following equation.

$$ Keratin yield = \frac{weight\, of\, chicken\, feather - weight\, of\, extracted\, keratin}{{weight \,of \,chicken\, feather}} \times 100 $$

#### Sodium dodecyl sulphate polyacrylamide gel electrophoresis (SDS-PAGE) analysis

Protein molecular weights were determined using a sodium dodecyl sulfate–polyacrylamide gel electrophoresis technique (SDS-PAGE). To dissolve the sample, SDS, dithiothreitol (DTT), glycerol, and bromophenol blue were used. The solution was heated in a boiling water bath for 5 min. Polyacrylamide 12% was used to stack and separate the gels. The electrophoresis voltage was (100–120) V (Supplementry data). After gel electrophoresis, the gel was washed with water and stained with a staining solution (Coomassie Brilliant Blue, water, methanol, and glacial acetic acid). After that, the sample was de-stained with a solution of water, methanol, and glacial acetic acid. A protein standard (Broad –Way Dual Prestained Protein Marker that resolved to 10 bands between 7 and 240 kDa.) was used for calibration Fig. S3.

#### Characterization of the extracted keratin

The structure and morphology of isolated keratin was analyzed using scan electron microscope (SEM) (JEOL JSM 6360LA, Japan), and FTIR was conducted using a Bruker Vector FTIR spectrometer to detect the protein's characteristic bonds.

#### Preparation of PAN, and PAN-keratin nanofiber by electrospinning

**Figure 1:**
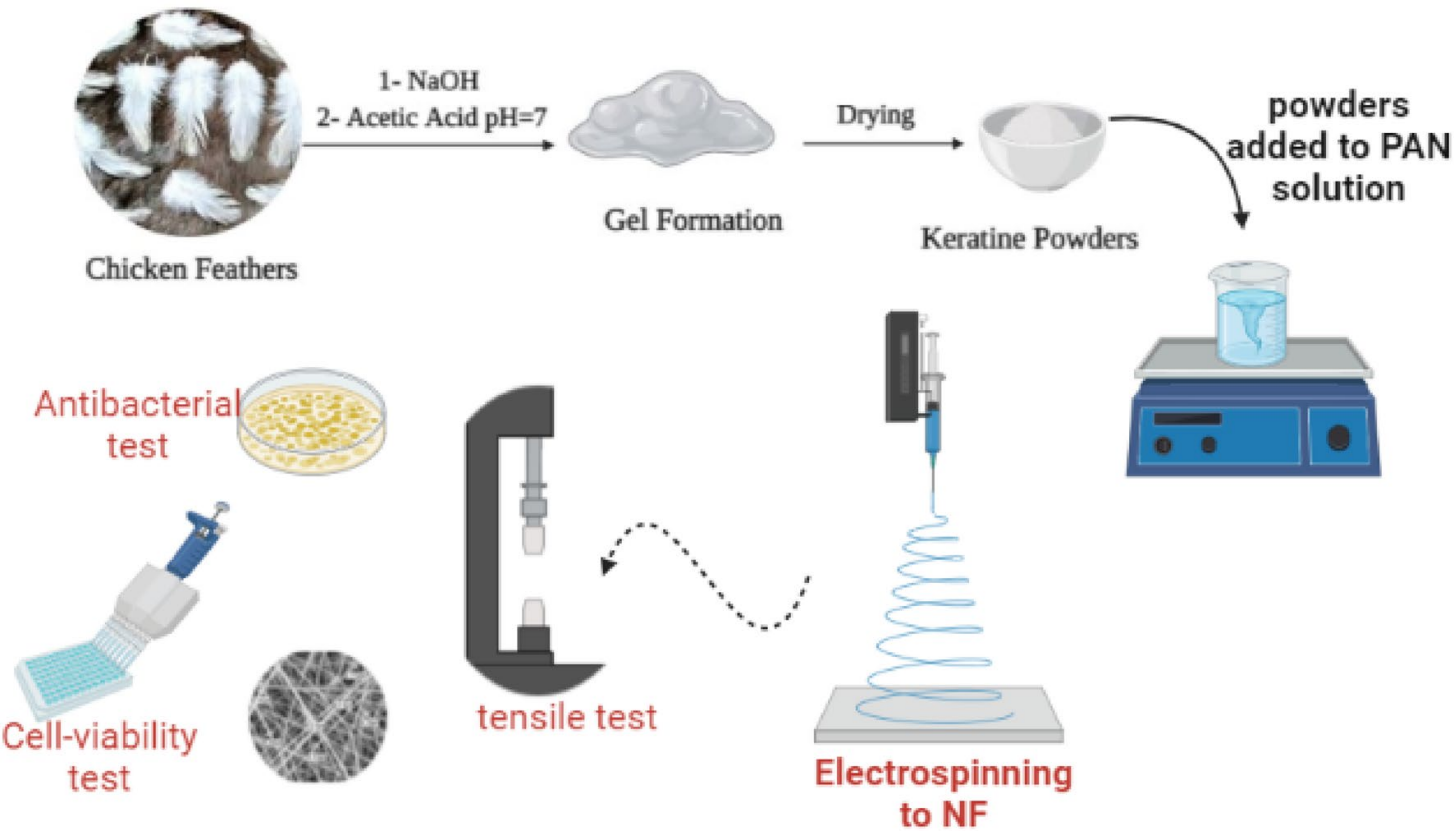
Illustrate the isolation of keratin, preparation of PAN/K nanofiber mats, and their characterizations. On the Biorender link https://app.biorender.com/illustrations/5de4cd57a20d3100816c6e05.

Electrospinning precursor solution PAN/DMF was prepared via dissolving (5.0% w/v) PAN in DMF, followed by the addition of different concentrations of feather keratin (0, 25, and 50) mg as shown in Fig. 1. The Keratin/PAN/DMF solution was loaded into a 10 ml syringe fixed on a syringe pump with a flow rate of 0.5 ml h^−1^, the spinning distance from the syringe to the collector was 10 cm, and the applied voltage was 20 kV.

#### Characterization of nanofibers mats

PAN, and PAN/Keratin nanofiber mats were investigated using a scanning electron microscope (SEM) (JEOL JSM 6360LA, Japan). The average nanofiber diameters were measured using the Smile View software with randomly selected SEM micrographs. The PAN/Keratin nanofiber mat were characterized by High resolution transmission electron microscope (JEOL 2100 PLUS, Japan). Transmission electron microscopy was used for the purpose of imaging, crystal structure revelation and elemental analysis “qualitative and semi-quantitative” analysis. Two different modes of imaging were employed; the bright field at electron accelerating voltage 200 kV using lanthanum hexaboride (LaB6) electron source gun and the diffraction pattern imaging. Eagle CCD camera with (4 k ∗ 4 k) image resolution was used to acquire and collect transmitted electron images. TEM Imaging & Analysis (TIA) software was used to spectrum acquisition and analysis of EDX peaks.

The chemical structures of isolated keratin, pure PAN nanofiber, and PAN/Keratin nanofibers (PAN/K) were conducted by FTIR-ATR spectroscopy. IR spectra were obtained also using a Bruker Vector FTIR spectrometer with a Helios ATR attachment containing a diamond crystal (≈ 250 μm × 250 μm sampling area) (Bruker Optics Ltd, Coventry, UK), Spectra. The ATR crystal was cleaned with distilled water and dried with dry tissue paper before the acquisition of spectral background. At room temperature, X-ray diffraction scans of nanofiber samples were obtained using the X-ray 7000 Shimadzu-Japan. By measuring the Bragg angle (2θ) in the range of 10 to 80 degrees, the degree of crystallinity of the prepared materials PAN and PAN/K nanofibers was determined. The X-ray source is a Cu target with a scan speed of 4 degrees per minute and a voltage of 30 kV and 30 mA. The tensile properties were studied using a universal testing machine (model AG-I, Shimadzu, Kyoto, Japan). The nanofiber mats were cut into rectangles with a size of 5 cm (length) × 5 cm (width) × 0.8 mm (thickness) and then conducted for tensile stress–strain curves.

#### Physiochemical characterization of the prepared nanofibrous mats

- Porosity test

The porosity of nanofiber mats was measured using the liquid displacement method at 37 °C^28^. Different weights of nanofibers (5, and 10) mg were submerged separately in distilled water for 24 h to achieve equilibrium. After that, the mats were spoiled with tissue paper, and the final weight was taken.

The porosity of the mats was calculated according to Eq. (1)

1 $$ Porosity \% = \left[ {\frac{{\left( {W_{2} - w_{1} } \right)}}{\rho Vs}} \right]*100 $$

W_1_ and W_2_ are the weights of the nanofibers before and after immersion in distilled water, respectively, ρ is the density of distilled water and Vs is the volume of the nanofibers.

- Swelling test

The weighted samples of nanofiber mats were submerged in water for a set amount of time at room temperature. After that, the samples are removed and blotted with filter paper before being weighted. For each sample, the swelling test was replicated 3 times. Equation (2) is used to calculate the swelling percent of each nanofiber mat.

2 $$ Swelling \% = \left[ {\left( {w_{s } - w_{d} } \right)/w_{s} } \right] \times 100 $$

where W_d_ is the weight of the hydrogel after soaking at a definite time interval and drying the gel, while W_s_ is the weight of the swollen hydrogel at a definite time.

- The roughness test

The surface roughness of the nanofiber mats was measured using a surface roughness tester (SJ-201P, Japan).

nanofiber mats with dimensions of 25 mm × 25 mm were fxed onto a glass slide using double-sided tape, and the measurements were carried out in three replicates (n = 3).

- Hemocompatibility test

Consistent with the American Society for Testing and Materials (ASTM F 756-00, 2000), the proposed hemolysis test is carried out^29^. Initially, 9 mL of blood were carefully poured into a tube containing 1 mL of anticoagulant (ACD).Each membrane (1 cm^2^) was submerged in a test tube containing 7 ml of phosphate buffer solution (PBS) pH 7.0 at 37 °C for 72 h prior to contact with rabbit blood. After discarding the PBS, the samples were immersed in 1 ml of ACD blood and kept at 37 °C for 3 h. Equal volumes of ACD blood were mixed with 7 mL of PBS and water, respectively, to create the negative and positive controls. The tubes were slightly inverted twice every 30 min to keep the examined materials from coming into contact with the blood. Finally, the blood and diluted blood were transferred to tubes and clarified by centrifugation at 2000 rpm for 15 min. A spectrophotometer set to ʎ = 540 nm was used to measure the liberated haemoglobin as a result of blood hemolysis. The blood hemolysis experiments were repeated three times under identical conditions, and the percentage of hemolysis was calculated using Eq. (3):

3 $$ Blood \,hemolysis \% = \left[ {\frac{ODs - ODn}{{ODp - ODn_{ } }}} \right] \times 100 $$

where ODs is the optical density of a tested sample, ODn refers to the optical density of the negative control, and ODp is the optical density of the positive control.

- Thrombogenicity test

A gravimetric method was used to assess the development of thrombus on the surface of the developed membranes, as previously proposed^30^. Mats were first immersed in PBS and incubated at 37 °C for 48 h. The PBS was then withdrawn, and the ACD rabbit blood was placed to the surface of the objects being investigated, with the same amount of blood added to an empty Petri dish as a positive control. 20 µl of a calcium chloride (10 M) solution was poured on top of the membrane to induce the blood clotting reaction.

After 45 min, the reaction was terminated by adding 5 mL of H_2_O. The clots were ultimately fixed with 5 mL of a formaldehyde solution (36%), then dried with tissue paper before being weighed. Three replicates of each test were supported.

- Antibacterial studies

For all samples, the antimicrobial activities of PAN and PAN/keratin membranes (PAN/0.025% K and PAN/0.05% K) were determined using an agar well diffusion assay^31^. Gram negative bacteria *Pseudomonas aeruginosa ATCC 27853* (*P. aeruginosa)* and Gram positive bacteria *Staphylococcus aureus ATCC 25923* (*S. aureus)* are two pathogenic microbial species that were used in this study. The bacteria were grown for 24 h in nutrient broth at 37 °C. The antimicrobial activity of three concentrations of reconstituted tested material against pathogenic strains was investigated. On agar media, 100 µl of inoculums (1108 cfu/ml) were inculcated on agar and poured into the Petri plate. With the help of a cork-borer (0.5 cm), a well was created in the plates, and 100 µl of the tested compound was poured into it. All of the bacteria were incubated for 24 h at 37 °C. The diameter of the inhibition zone around the well (mm), including the well diameter, was used to calculate the zone of inhibition. In all three triplicates, readings were taken in three different fixed directions, and the average values were tabulated.

The minimum concentrations of antibacterial keratin necessary to inhibit bacterial growth (MIC) were determined against *P. aeruginosa* and *S. aureus* after 24 h of incubation at 37 °C in nutrient broth. Based on the previous results in the current study, a PAN/0.05% K nanocomposite sample at a keratin concentration of 12.5 µg/mL was selected as the initial concentration for conducting the antibacterial test. Serially diluted in saline and plated on a nutrient agar plate, 100 µl of inoculums (1108 cfu/ml) were inculcated on agar and poured into the Petri plate, and incubated for overnight. In parallel with PAN/K samples, ampicillin, as a positive control, was tested as a therapeutic agent used in the treatment of infections. The minimal inhibitory concentration (MIC) values were determined in triplicate using a microplate serial dilution technique, and the mean values were reported.

#### The safety assay of PAN and PAN/K nanofiber mats

The safety assay was performed for determination of treatments concentration that does not record the IC_50_ on the non-cancerous cell line (WISH cells). 100 µl of 6 × 10^4^ cell/ml cells were seeded in 96-well plates, and the plates were incubated at 37 °C in humidified 5% CO_2_ for 24 h. After a semi confluent, the exhausted old (DMEM) medium was replaced with 100 µl of different treatment concentrations. The treated plates were incubated at the same growth conditions for 24 h. At the end of incubation, cellular viability was quantified using MTT assay protocol according to the manual instruction.

#### Cell proliferation assay

*Preparation of nanofibers samples for cell culture*: The samples were sterilised with 70% ethanol for 30 min before being washed three times with sterile PBS, immersed in a culture medium for 30 min, and then placed in a 96-well plate (Nunc, Wiesbaden, Germany) for cell culture experiments.

MG-63: Osteosarcoma was obtained from Nawah Scientific Inc.,(Mokatam, Cairo, Egypt). Cells were maintained in DMEM media supplemented with 100 mg/mL of streptomycin, 100 units / mL of penicillin and 10%of heat-in activated fetal bovine serum in humidified, 5% (v/v) CO_2_ atmosphere at 37 °C.

Cell viability was assessed by WST-1 assay using Abcam ® kit (ab155902 WST-1 Cell Proliferation Reagent). Aliquots of 50 μL cell suspension (3 × 10^3^cells) were seeded in 96-well plates and incubated in complete media for 24 h. Cells were were cultured for each 6.0 mm diameter rounded sample (n = 3). After 48 h of drug exposure, cells were treated with 10μL WST-1 reagent and the absorbance was measured after 1 h at 450 nm using aBMGLABTECH ®-FLUO star Omega microplate reader (All mendgrün, Ortenberg).

The percentage of viable cells was calculated using the following formula:

4 $$ Cell \,availability = \frac{{ A_{{\exp - A_{control} }} }}{{A_{Postive} - A_{control} }} $$

where A exp represents the test sample's experimental absorbance (nanofibrous samples + cell), A control represents the blank sample absorbance (media without cells), and A positive represents the positive control absorbance (media + cells).

### Statistical analysis

Each experiment was repeated at least three times and each test group had at least quadruplicate repeats. The average and standard error of the mean were calculated from the results of all experiments (SEM). The data were analysed using one-way analysis of variance (ANOVA) through Minitab 16 software, with a significance level of at least *p* < 0.05.

## Results and discussion

The extraction rate of Feather keratin isolation was 78.4% when sodium hydroxideNaOH was used as an extractant, and it was found to be capable of breaking hydrogen bonds and introducing electrostatic repulsion via charged residues, resulting in increased solubility. This ratio is comparable to that of other stuides that used sodium hydroxide and sodium bisulphite as extraction solvents on the keratin extraction, with yield after validation of 68.3% and 65.2%, respectively^32^. Otherwise, Pourjavaheri and colleagues used sodium sulphide and l-cysteine as extractants, with a yield of extracted keratin of 88% and 66%, respectively^33^.

**Figure 2:**
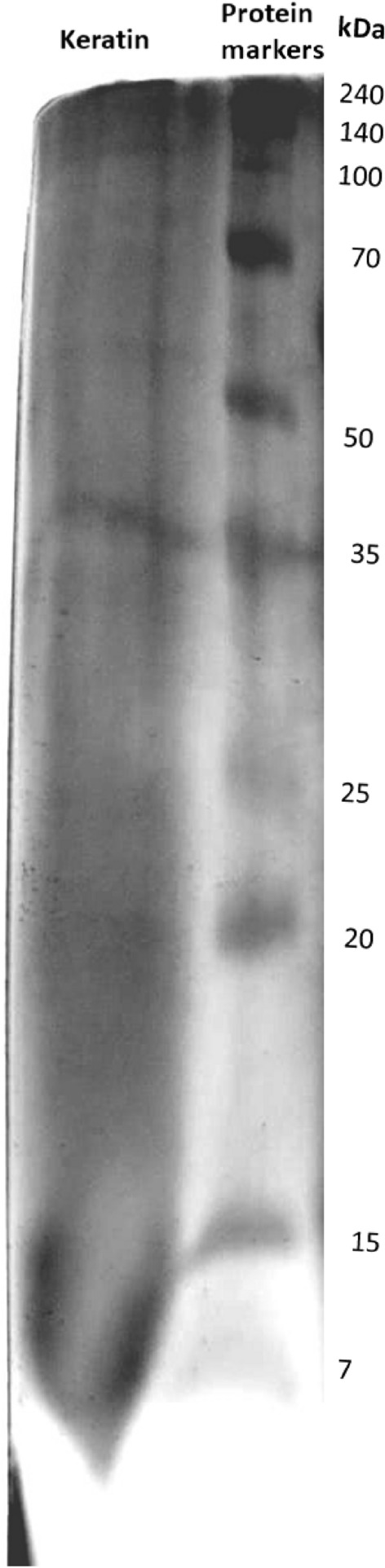
SDS-PAGE of the extracted feather keratin.

SDS PAGE electrophoresis of the extracted keratin is plotted by Image J 1.53i software in Fig. 2, and a protein band in 35 kDa is observed, indicating that the regenerated keratin had a molecular weight of 35 kDa. The major rotein bands with molecular masses ranging from 40 to 60 kDa are microfibril keratins of α keratin^34,35^.

**Figure 3:**
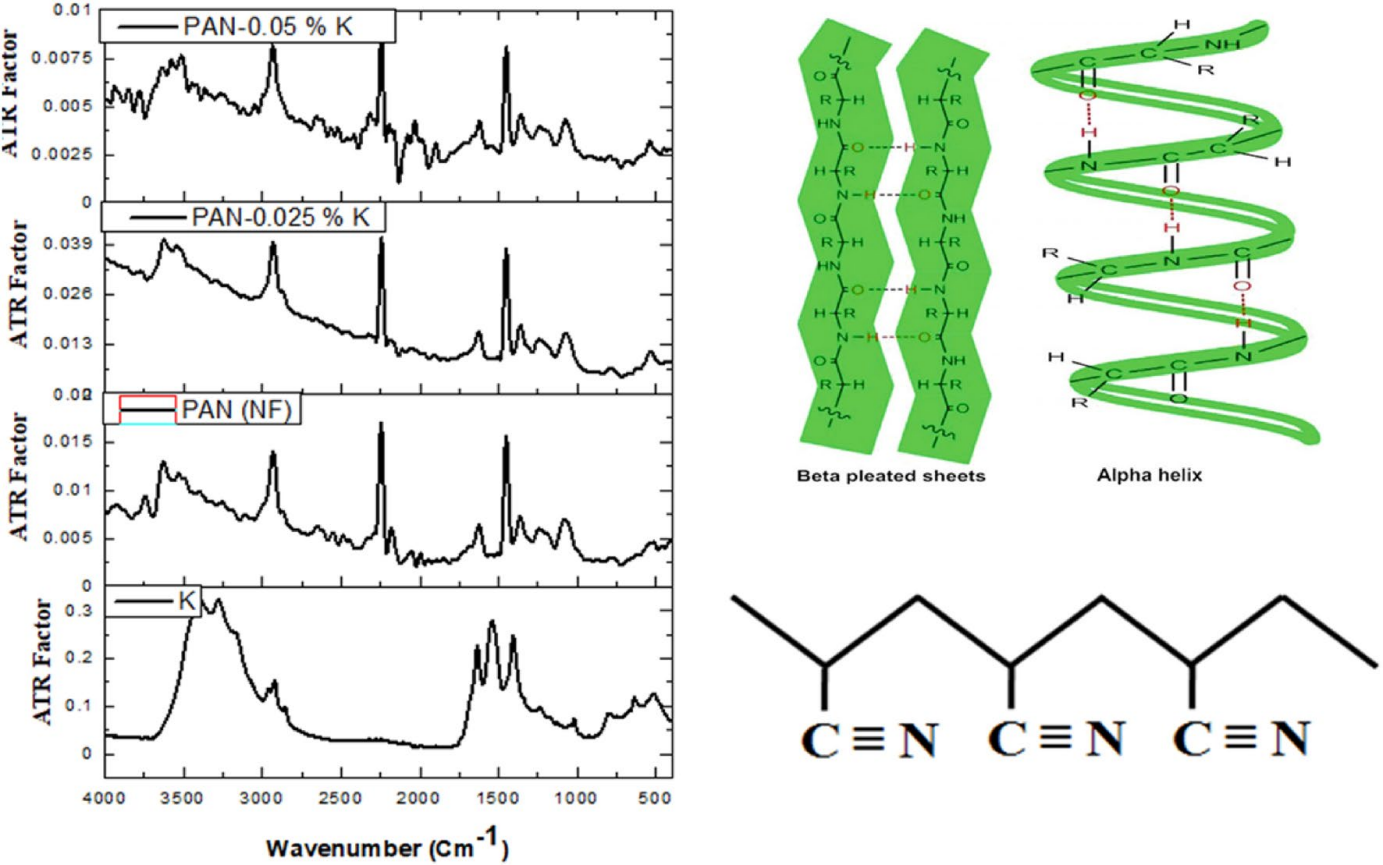
FTIR-ATR spectrum of the prepared 4 samples (isolated feather keratin (K), Pure PAN nanofiber (PAN (NF)), and PAN/0.025% K, and PAN/0.05% K), with the chemical structures of keratin (upwards-right side) and polyacrylonitrile (downwards-right side).

The chemical composition and conformational changes of isolated keratin, PAN nanofiber, and PAN/Keratin nanofibers (PAN/0.025% K, and PAN/0.05% K) were investigated using FTIR-ATR analysis as shown in Fig. 3, and Table S1. The spectrum peak of keratin includes a strong transmission band at 3440 cm^−1^ assigned to the peptide bond (CO–NH)^36,37^, and a small band at 2935 cm^−1^ referred to the (CH_3_ group)^38^, while the characteristic peaks of amide groups I, II, and III for the keratin-sheet appear strongly at 1649 cm^−1^, 1556 cm^−1^, and 1247 cm^−1^, respectively. Where the vibration band of C=O refers to Amide I, it appears at 1646 cm^−1^^39^, the N–H bending and C–H stretching, which are related to Amide II, occur at wavelengths at 1580 cm^−1^^16^, otherwise, C–N stretching and N–H vibration, which are associated with Amide III, appear at 1300 cm^−1^^40^. PAN had characteristic peaks at 2239 cm^−1^ and 2946 cm^−1^ that were associated with C–N and CH_2_ stretching vibrations, which are distinctive chemical groups in PAN^41^. Functionalization of nanofibers mats with keratin lead to appearing peak at 1458 cm^−1^ is attributable to amide II, which has been linked to keratin N–H bending and C–H stretching^42^. Otherwise, the ATR-FTIR peaks at 550 cm^−1^ for PAN/0.025% K and PAN/0.05% K are associated with S–S of the disulfid bond of keratin^43^. Moreover, the peak at 1065 cm^−1^ appears to be stronger on PAN/Keratin spectrum than on PAN curves, which is due to alkyl C–N vibration which associated with Amide III of keratin, which proves the successful reinforcing of keratin into the PAN nanofiber^44^.

**Figure 4:**
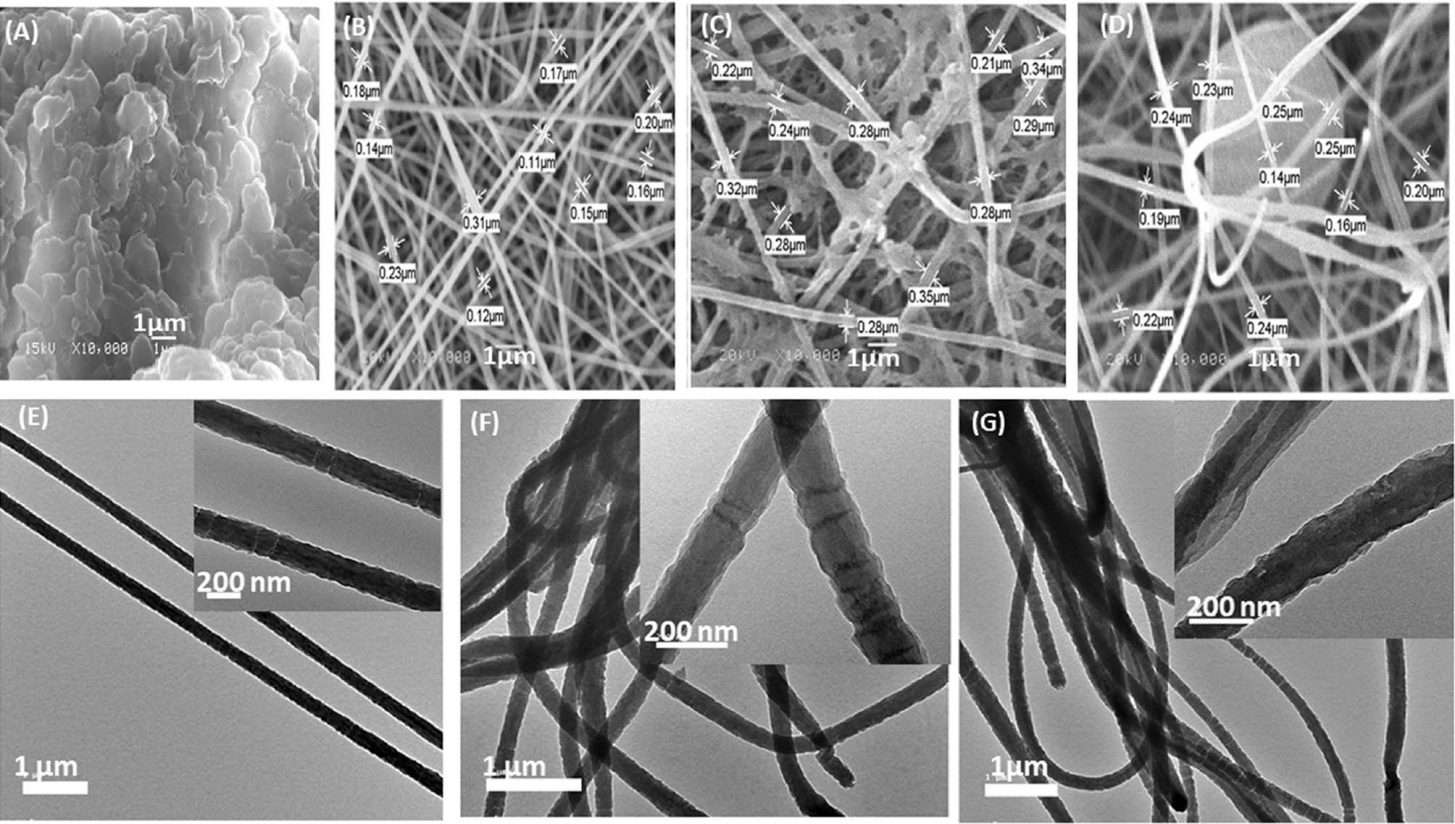
The SEM images at magnification(1 µm) for the isolated keratin, [pure PAN, PAN/0.025% K, and PAN/0.05% K] nanofibers, are shown in (**A**), (**B**), (**C**), and (**D**). The TEM images with low magnification (1 µm) and high magnification (200 nm) of pure PAN, PAN/0.025%K, and PAN/0.05%K are shown in (**E**), (**F**)and (**G**), TEM images have been developed by MINITAB 16.1(Mtb.exe). http://www.minitab.com/en-us/.

The morphology of isolated keratin and nanofiber mats via SEM and TEM is shown in Fig. 4. The mats were constructed from randomly aligned nanofiber mats. PAN nanofibers possess a smooth, uniform surface with a diameter of 177 nm. Electrospun PAN/0.025K nanofiber mats, on the other hand, had rough surfaces, whereas PAN/0.05K nanofiber mats were smoother and had a more uniform structure than PAN/0.025K nanofiber mats. This can be explained by the fact that adhesion occurred at low keratin concenteration, but increasing keratin concenteration caused inter-intra melcular interation between many keratin function groups (amide and arboxylic groups) and PAN, and this result was similar to previous studies^45–47^ in which nanofiber mats became more uniform in shape and thinner as the portions of keratin increased. Furthermore, the surface of PAN nanofibers was coated with keratin, which could help improve nanofiber biocompatibility^48^. This is similar to the coating of polycaprolactone (PCL) nanofiber with alternating layers of positively and negatively charged gelatin, the protein being electrostatically stacked on the NFs^49^. Furthermore, Noishiki and colleagues coated a polymer stent with a heparinized keratin derivative and implanted it in a dog for more than 200 days without thrombosis. In the same year, polyester meshes were coated with keratin and implanted into rabbit and dog dorsal muscles, and this led to an increase in the biocompatibility of the implanted filaments^50^. The fiber's average diameter increased from 177 nm for PAN nanofiber to 212 nm for PAN/0.025 Keratin nanofiber and to 280 nm for PAN/0.05 Keratin nanofiber, fiber diameters were determined by using smile view Map software with the link https://www.jeol.co.jp/en/products/detail/SMILE_VIEW_MAP.html. This result is in line with the calculated porosity value of the PAN nanofiber and PAN/ Keratin nanofiber mats as shown in Table 1. The addition of keratin to nanofiber mats increases their porosity, which is beneficial for wound healing because the porous structure of the mats allows vital nutrients to reach the cells and allows gas and fluid exchange at the wound site^30^. Moreover the porosity of the wound dressing can have a significant influence on the exudate absorption capacity, lowering the risk of wound infection^51^.

**Table 1:** Porosity % with tensile properties of electrospun fibers.

Nanofiber mats	Roughness (µm)	Porosity (%)	Mechanical tests		
			Tensile strength (N/mm^2^)	Strain at break (%)	Young Modulus (N/mm^2^)
PAN	0.1365 ± 0.034	24.52 ± 2.12	0.738	0.0140	0.0189
PAN/0.025 K	0.149 ± 0.045	32.55 ± 1.27	0.541	0.0093	0.0172
PAN/0.05 K	0.223 ± 0.008	90.89 ± 1.91	0.781	0.0234	0.0299

The elemental composition of composite nanofibers was investigated using EDX as shown in Table 2, and Figure S1. At low keratin concenteration, PAN/0.025K nanofiber was primarily composed of C and O. This outcome was comparable to that of the FK/PVA/PEO composite nanofiber^52^. Shankar and colleagues also used an EDX elemental analyzer to examine the elements of keratin nanoparticles and their metal composite (KNP and KNP-metal ion complexes), and KNP revealed carbon and nitrogen peaks for the keratin protein backbone. KNP revealed keratin protein backbone carbon and nitrogen peaks. While the EDX peaks of the KNP-Ag, KNP-Cu, and KNP-Zn complexes showed carbon and nitrogen peaks in addition to silver, copper, or zinc peaks, KNP and its complexes seemed to have no sulphur content^53^. The sudden appearance of sulphur in PAN/0.05K nanofiber was attributed to cysteine amino acids of keratin, which form intermolecular and intramolecular disulfide bonds and are responsible for the protein's high stability^20^.

**Table 2:** EDS of pure PAN, PAN/0.025% K, and PAN/0.05%K.

Weight%	PAN	PAN/0.025% K	PAN/0.05%K
C	94.62	98.66	99.74
O	5.38	1.34	–
S	–	–	0.26

**Figure 5:**
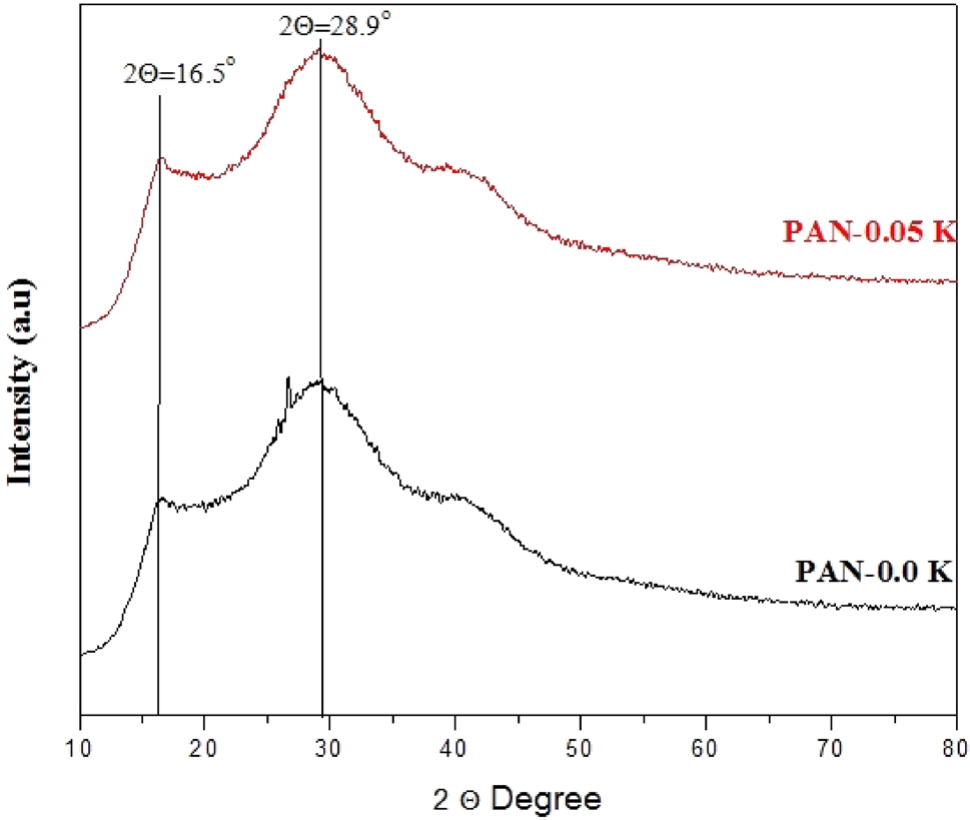
XRD pattern of pure PAN nanofiber mat, and PAN/0.05% K nanofiber mats.

Figure 5, S2 shows an XRD pattern of pure PAN and PAN/0.05% K nanofiber mats, with two broad peaks at 2θ (16.5° and 28.9°) for both PAN and PAN/0.05% K. These peaks are typical of PAN nanofibers^54,55^. According to the observed diffraction peaks, the addition of keratin at that low concentration had no effect on the crystallinity of PAN. The intensity of diffraction showed that the blend nanofibers had a degree of crystallinity similar to that of the electrospun PAN fiber, and this result matched the result of blending keratin with PVA nanofiber in previous studies^56^.

**Figure 6:**
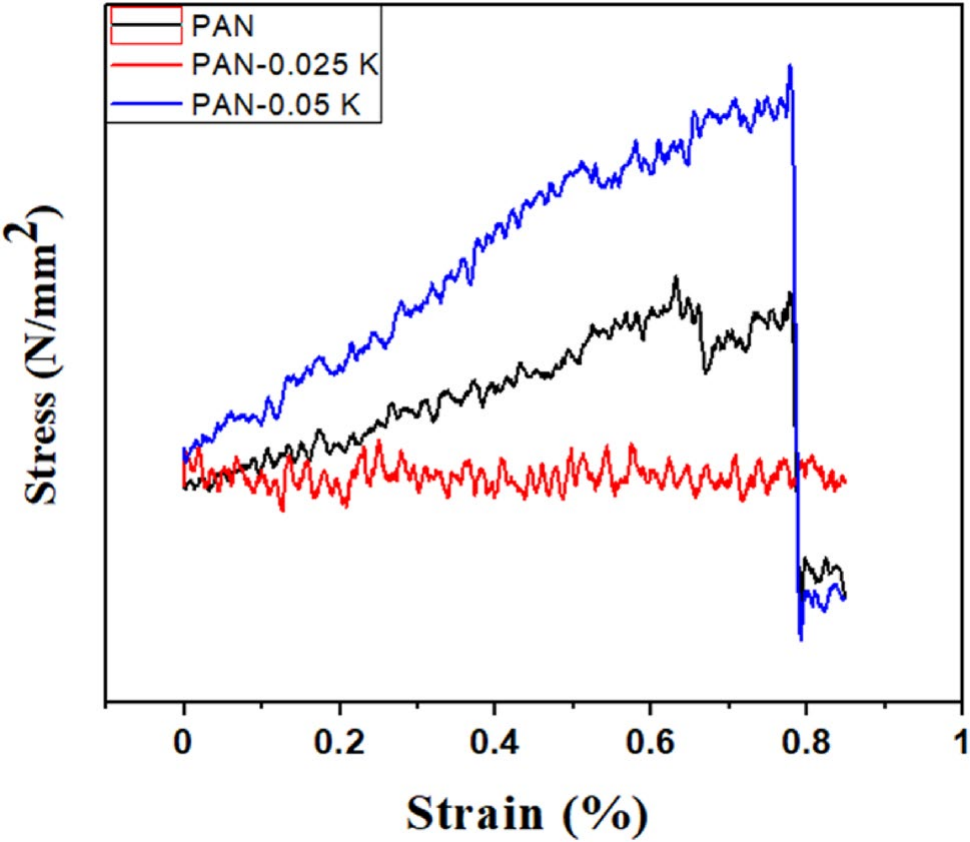
Representative stress–strain curves of pure PAN, PAN/0.025% K and PAN/0.05% K nanofibers mats.

Strength and flexibility are the most important factors to consider when applying wound dressings. An ideal wound dressing should be strong enough to withstand external impacts but flexible enough not to obstruct movement or damage the tissue on which it is applied. Figure 6 shows the stress versus strain curves for pure PAN and the PAN/K nanofibers mats with different keratin contents. The average tensile strength (N/mm^2^) for pure PAN, PAN/0.025% K, and PAN/0.05% K were 0.738, 0.541, and 0.781 respectively. The average Young modulus (N/mm^2^) were 0.0189, 0.0172, and 0.0299 respectively. The results showed that the addition of 0.025% keratin changed the mechanical properties of the nanofibers, causing them to have lower tensile strength, and elongation at break than pure PAN nanofibers. This may be due to the agglomeration of the keratin nanoparticles throgh the fiber threads as shown in Fig. 4c. While the tensile strength increased as the keratin content increased (0.05%). This tendency was observed in previously studies keratin/PEO nanofiber mats^57^ and wool keratin/PVA nanofiber mats^56^; the addition of keratin with low ratios made the nanofibers stiff, whereas the tensile at break was slightly improved as the keratin ratio increased. The mechanical properties and bond lability of keratin-based materials have been revealed, and many of the reinforcing mechanisms have previously been discussed. Keratins were divided into two groups based on their structure, function, and regulation: (1) “Hard” keratins (alpha chain), which form ordered filaments embedded in a cysteine-rich protein matrix and have a compact and hard structure; (2) “Soft” keratins (beta chian), which form loosely-packed bundles of filaments and have the function of granting elongation and stress release. Compared to alpha-keratin amino acid chains, beta-keratin amino acid chains are shorter. For instance, only 32 amino acids make up the central rod domain, 23 amino acids make up the head domain, and 47 amino acids make up the tail domain in the beta-keratin of feather, whereas alpha-keratins can contain hundreds of amino acid residues. Under mechanical stress, keratin showed a transition from a central alpha helical coiled coil rod (stiff) to an elongated beta strand structure (elastic)^58^. Additionally, the mechanical characteristics of the synthesised nanofibers may be increased due to the keratin's interfacial adhesion to the PAN matrix and the intermolecular hydrogen bonding that takes place between backbone molecules and adjacent chains of the "sheet of feather" keratin (in either a parallel or anti-parallel direction)^59,60^.

**Figure 7:**
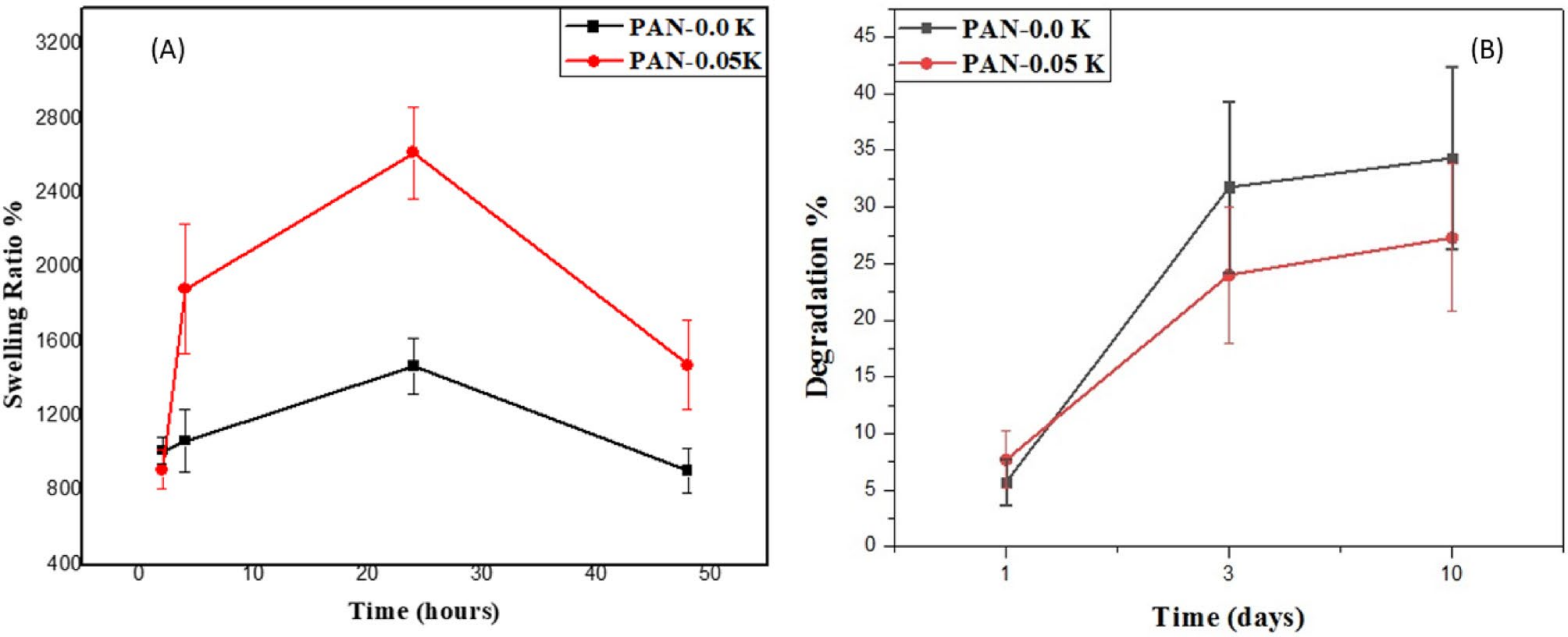
The swelling and biodegradation of PAN, and PAN/0.05%K nanofiber mats (A, and B) respectivelly.

The ability of electrospun non-woven mats to absorb wound exudates produced during the inflammatory phase of the healing process is determined by their swelling ratio. To do so, the PAN and PAN/0.5% K samples were incubated in a PBS solution at 37 °C for 50 h before being weighted. As shown in Fig. 7, the keratin-based sample had a significantly higher swelling ratio when compared to the control PAN electrospun mat. This behavior can be explained by the higher porosity and specific surface area of keratin-based electrospun mats compared to the pure PAN one, and the highly keratin polar nature, which allows the protein to form stable complexes with water molecules^61^.

### The wound dressing evaluation

#### Hemolysis assay

**Figure 8:**
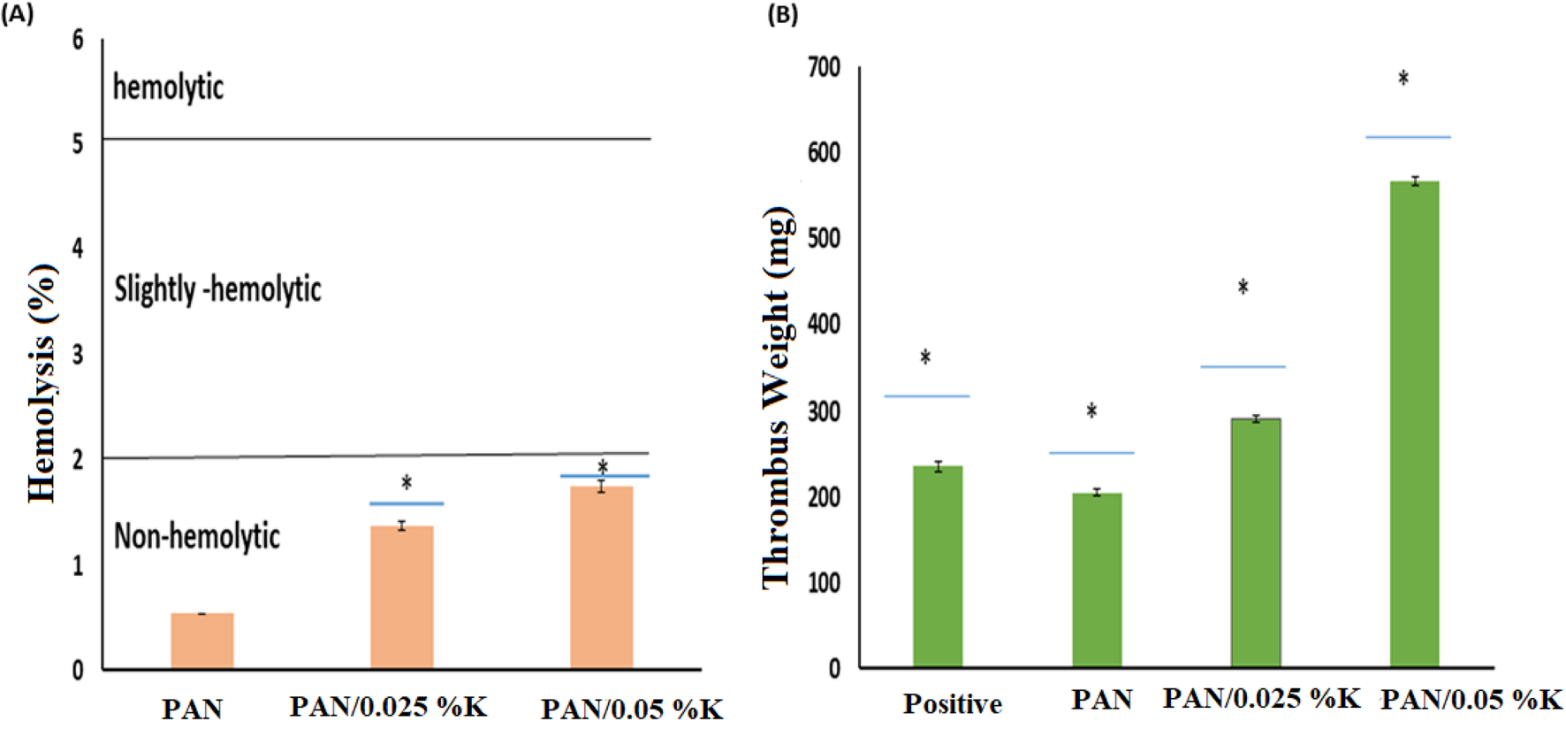
(**A**) Hemocompatibity, (**B**) and thrombogenicity for PAN nanofiber membrane and PAN/K nanofiber membranes. Data are expressed as means ± SD (*p < 0.05).

Blood compatibility must be assessed for biomaterials that come into direct or indirect contact with blood. The hemolysis test is an important *in-vitro* rough screening and blood compatibility evaluation test. Figure 8a shows the hemolytic percentages of the nanofiber mats studied. The findings revealed a slight increase in hemolytic efficiency between modified and unmodified mats. However, the percentage of hemolysis in all prepared samples is still less than 2%, which is considered safe by ASTM F 756-00, 2000, and these findings are consistent with previous research^29^, and these results are in line with the results reported in previous studies^54,56^. PAN/keratin nanofiber mats were found to be compatible with non-hemolysis materials.

#### Thrombogenicity assay

The material's thrombogenicity is an important property for wound healing. Figure 8b shows that PAN nanofiber had a lower tendency for thrombus formation than PAN/keratin nanofiber, which could be attributed to keratins' intrinsic ability to induce integrin-mediated platelet activation and adhesion^18,19,62^.

As a result, PAN/keratin nanofibers were an excellent biodressing material that kept the wound moist while maintaining mechanical strength during healing.

#### Antibacterial studies

**Figure 9:**
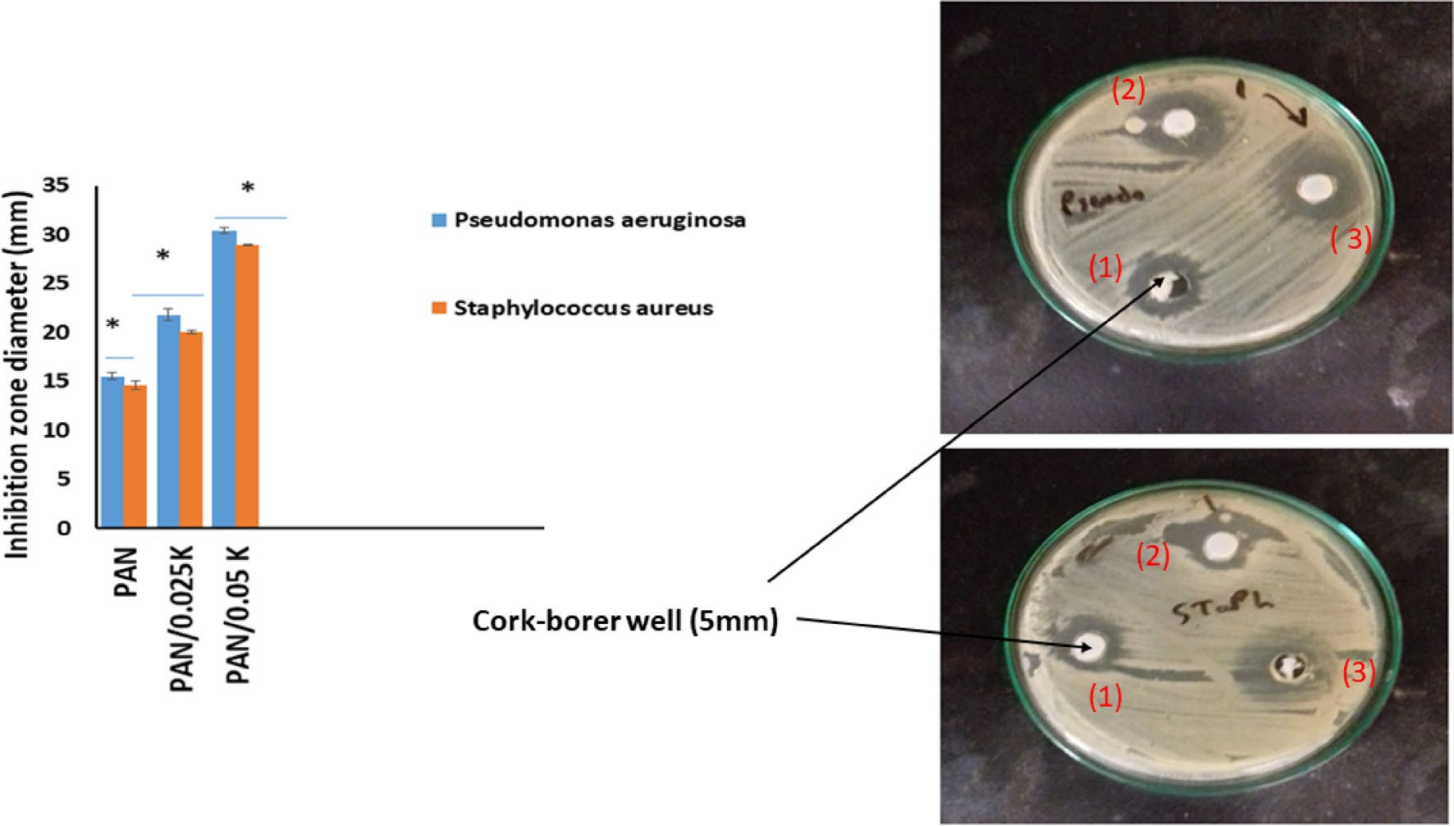
Antibacterial assays of PAN nanofiber membrane (labeled with 1), PAN/0.025K (labeled with 2) and PAN/0.05K nanofiber mats (labeled with 3) against *Pseudomonas aeruginosa, and Staphylococcus aureus*, Cork-borer well (5 mm). Data are presented as means ± SD (n = 3, *p < 0.05).

Feather keratin-loaded electrospun mats should be able to limit the growth of bacterial strains that cause serious wound infection, facilitating wound healing. The bacterial inhibition was examined using the zone of inhibition method as illustrated in Fig. 9. Gram negative bacteria (*P. aeruginosa*) and gram positive bacteria (*S. aureus*) are used to investigate the antibacterial activity of PAN and PAN/keratin nanofibers. PAN nanofiber showed a weak inhibition zone compared with that loaded with keratin. The best antibacterial activities were observed against *P. aeruginosa* (30 ± 0.17 mm) and *S. aureus* (29 ± 0.31 mm) at PAN/0.05% keratin. Therefore, the antibacterial effect has a direct relationship with keratin concentration, and this result is in line with^63–65^.

The minimum inhibition concentrations (MICs) of different concentrations of keratin in PAN nanofiber and control antibiotic (0.1%) against *S. aureus* and *P. aeruginosa* are reported in Table 3. The MIC concentration of keratin in PAN nanofiber for both *S. aureus* and *P. aeruginosa* in the current study was 3.12 µg/mL. As shown in Table 3, the next concentration of keratin (1.56 µg/mL) give the same inhibition zone of zero conceneration of keratin (15 mm for *P. aeruginosa, and 14 mm for S. aureus, respectively),* so this concentration couldn’t express antibacterial effect.

**Table 3:** Minimum inhibition concentration of keratin in PAN nanofiber µg/ml.

Pathogenic strain		Concentration of keratin in PAN nanofiber µg/ml				
	Antibiotic (0.1%)	4.68	3. 12	1.56	0	MIC
		Inhibition zone diameter (mm)**				
Pseudomonas aeruginosa ATCC 27853	45	20	17	15	15	3.12
Staphylococcus aureus ATCC25923	27	18	16	14	14	3.12

Otherwise, Studies on the antibacterial properties of keratinous materials (either extracted from wool, chicken feathers, or human hair) had not yielded conclusive results in the recent literature. Caven et al.^66^, for example, looked into the antibacterial properties of wool against S. aureus. Other studies, on the other hand, found no evidence of strong bactericidal activity in keratin-based composite films and nanoparticles^67^. As a result, various interpretations of keratin macromolecules' antibacterial activity have recently been proposed, based on the presence of disulfide bonds and the content of coiled-coil random domains^67^, or depending on the amount of reduced protein molecules and the extraction methods used^68^.

#### Safe assay of PAN, and PAN/keratin nanofiber mats

**Figure 10:**
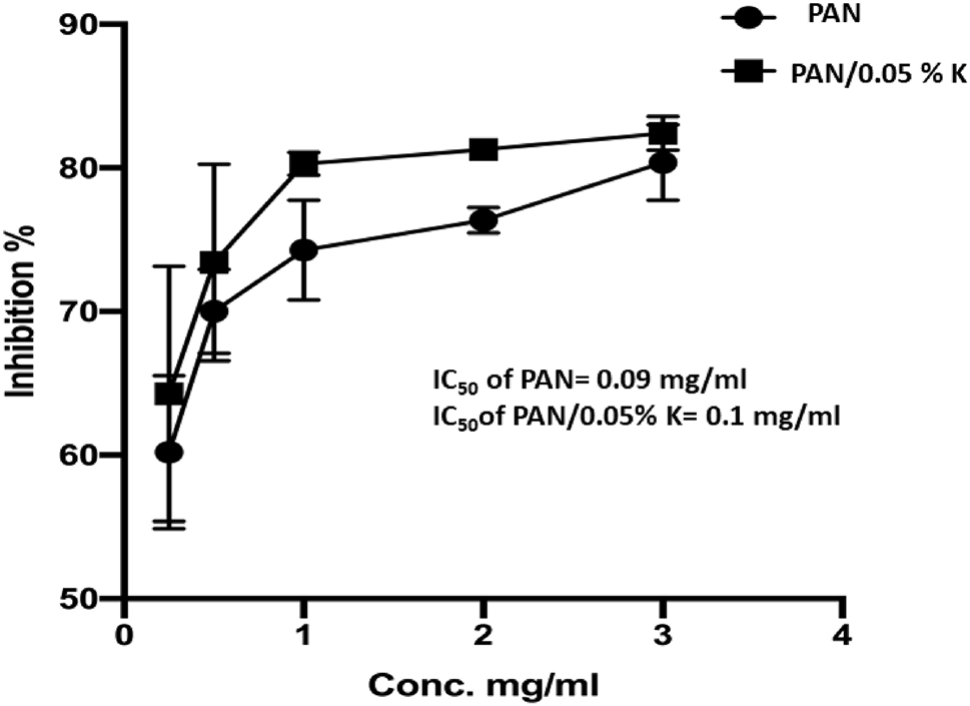
The inhibition % of WISH cells via PAN, and PAN/0.05%K. Data are presented as means ± SD (n = 3, *p* < 0.05).

The materials' cytotoxicity is critical for their wound-dressing applications. The MTT proliferation assay was used to assess the relative cell inhibition of extracts from the PAN and PAN/0.05% K Nanofibrous mat as shown in Fig. 10. After 24 h of incubation with the nanofiber mats, there was a significant reduction in WISH cell growth. In comparison to pure PAN nanofiber mats, PAN/0.05% K nanofiber mats showed high inhibition (80–82%) at 2 and 3 mg/mL concentrations. PAN and PAN/0.05% K, on the other hand, had IC_50_ values of 0.09 and 0.1 mg/ml, respectively. From these findings, it can be concluded that the nanofiber mats are well tolerated and do not irritate the skin. The cell viability was studied by the WST test. The WST test showed that the cell viability percentage increased by about 11% for PAN-0.05K when compared to the pure PAN membrane after two days of cell culture. Besides, the cell viability percentage was calculated by Eq. (4), as shown in Table 4.

**Table 4:** Cell viability percentage of the prepared samples after two days.

Samples	PAN	PAN-0.05K
Cell viability	85.71 ± 2.04	95.88 ± 1.88

## Conclusion

Keratin biomaterials have several distinct advantages over conventional biomolecules, including a unique chemistry provided by their high sulphur content, which varies from 6.34 to 94.47% depending on the exceptional biocompatibility, self-assembly proclivity, and intrinsic cellular functionality. To make PAN/K nanofiber mats, feather-based keratin was extracted first and then coelectrospun with polyacrylonitrile. Adding keratin to them increased their porosity from 24.5 ± 2.12% for PAN to 90.89 ± 1.91% for PAN/0.05% K due to the properties of keratin. The mechanical properties of the mats are improved by keratin as well. These mats had antibacterial properties without causing hemolysis or cytotoxicity (less than 2% hemolysis). At PAN/0.05% K, the best antibacterial activities were observed against Pseudomonas aeruginosa (30 ± 0.17 mm inhibition zone) and Staphylococcus aureus (29 ± 0.31 mm inhibition zone) bacteria. The addition of keratin to nanofiber mats resulted in the creation of nanocomposite mats with medical applications in mind, such as vascular tissue engineering scaffolds and chronic wound dressings.

## Supplementary Information

Supplementary Information.

## Data Availability

All data generated or analysed during this study are included in this article.
