# Supplementary material for: Electrospun non-wovens potential wound dressing material based on polyacrylonitrile/chicken feathers keratin nanofiber
Source: Sci Rep. 2022 Sep 14;12:15460. doi: 10.1038/s41598-022-19390-3 (PMC9474820; doi:10.1038/s41598-022-19390-3)
Supplement: Supplementary file 1 — Supplementary Information. [file 41598_2022_19390_MOESM1_ESM.docx]

**Electrospun Non-Wovens Potential Wound Dressing material based on Polyacrylonitrile / Chicken Feathers keratin Nanofiber**

**Eman Serag^1*^, Asmaa M. Abd El-Aziz^2^, Azza El-Maghraby^2^, Nahla A. Taha^3^**

**1** Marine Pollution Department, Environmental Division, National Institute of Oceanography and Fisheries, Kayet Bey, Elanfoushy, Alexandria, Egypt.

**2** Fabrication Technology Research Department, Advanced Technology and New Materials Research Institute (ATNMRI), City of Scientific Research and Technological Applications (SRTA-City), Alexandria, Egypt.

**3** Modeling and Simulation Research Department, Advanced Technology and New Materials Research Institute (ATNMRI), City of Scientific Research and Technological Applications (SRTA-City), Alexandria, Egypt.

**Coresponding authors:**

**Eman Serag^1*^,** [**d.emanserag@yahoo.com**](mailto:d.emanserag@yahoo.com)

**Asmaa M. Abd El-Aziz^2*^,** [**aabdelaziz@srtacity.sci.eg**](mailto:aabdelaziz@srtacity.sci.eg)

**Methods**

**Protocol of SDS-PAGE Technique:**

1. Let Broad-Way Multi Prestained Protein Marker adjust to room temperature before use. After completely thawing, mix them thoroughly.
2. For mini-gel load 5-10 ml to the bottom of the wells by the pipettor. For full-size gel (ex. 16 x 20 cm) load 10-15 ml.
3. Load samples.
4. Connect the power supply and start electrophoresis

**8-20% SDS-PAGE Blot**

Apply 5 ml on mini-gel and electrophoresed for 1hr at 15% SDS-PAGE gel. After electrophoresis, transfer for 2hr at 80 volt. [1]

**Results and Discusssion**

**Table S1. Major bands were observed in FTIR-ATR data.**

| Peak Number | Wavenumber (cm^-1^) | Functional groups |
| --- | --- | --- |
| 1 | 3440 | CO-NH *(K)* |
| 2 | 1649 | C=O of amide I *(K)* |
| 3 | 1580 | N-H of amide II *(K)* |
| 4 | 1300 | C-N of amide III *(K)* |
| 5 | 2233 | C-N stretching *(PAN)* |
| 6 | 2926 | CH_2_ stretching *(PAN)* |
| 7 | 1064 | alkyl C-N *(PAN / K)* |
| 8 | 1458 | keratin N-H bending and C-H stretching *(PAN / K)* |
| 9 | 550 | S-S of keratin disulfied bond *(K, PAN/K)* |


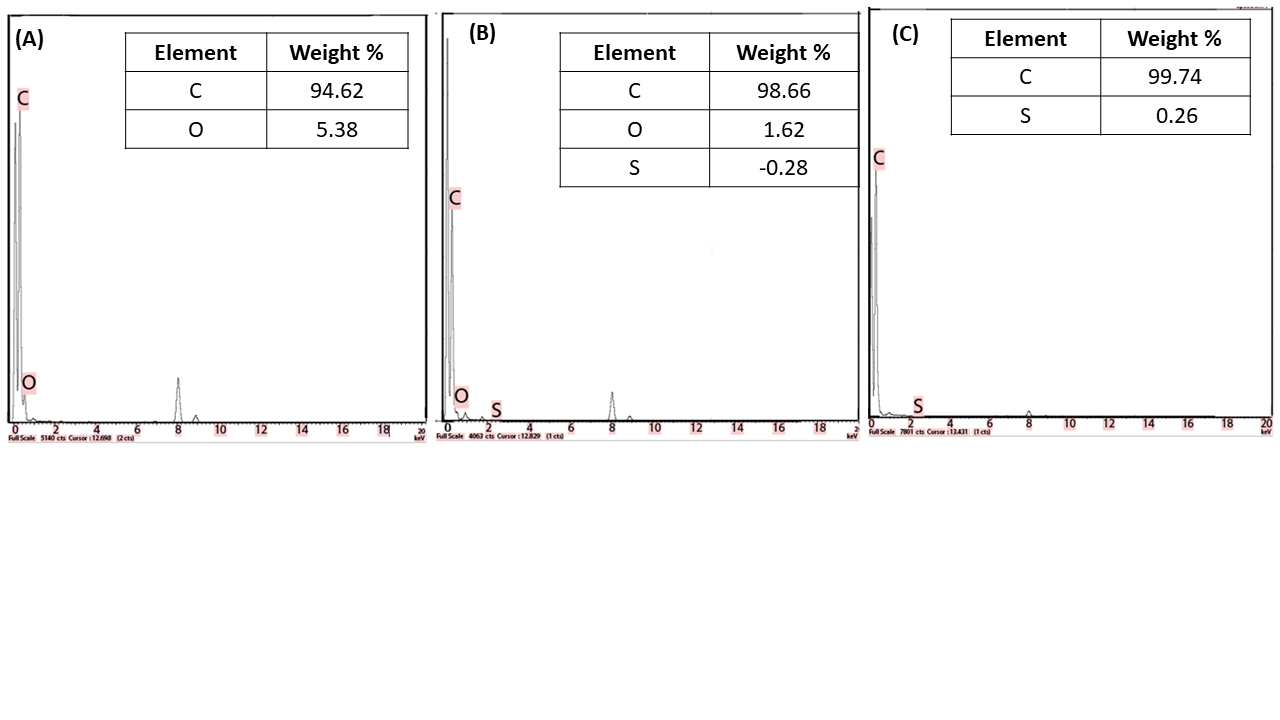


**Figure S1. EDS of pure PAN, PAN/0.025 % K, and PAN/0.05 %K are shown in (A), (B), and (C).**


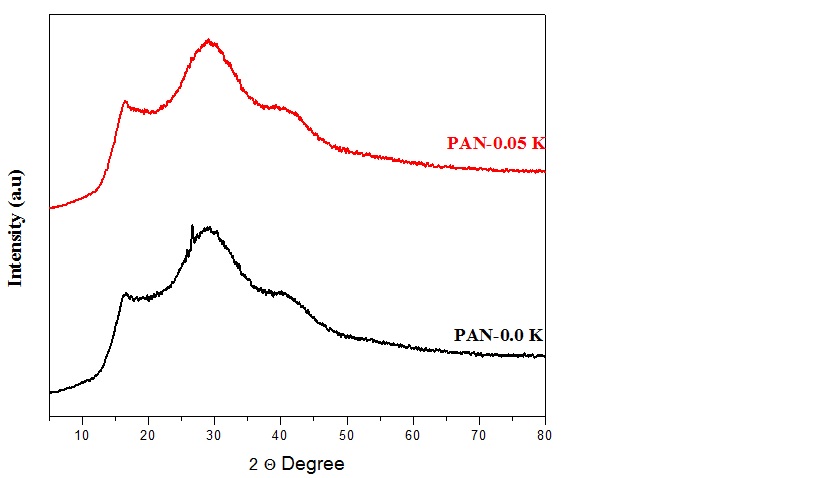


**Figure S2. XRD pattern of pure PAN nanofiber mat, and PAN/0.05% K nanofiber mats (full scale).**


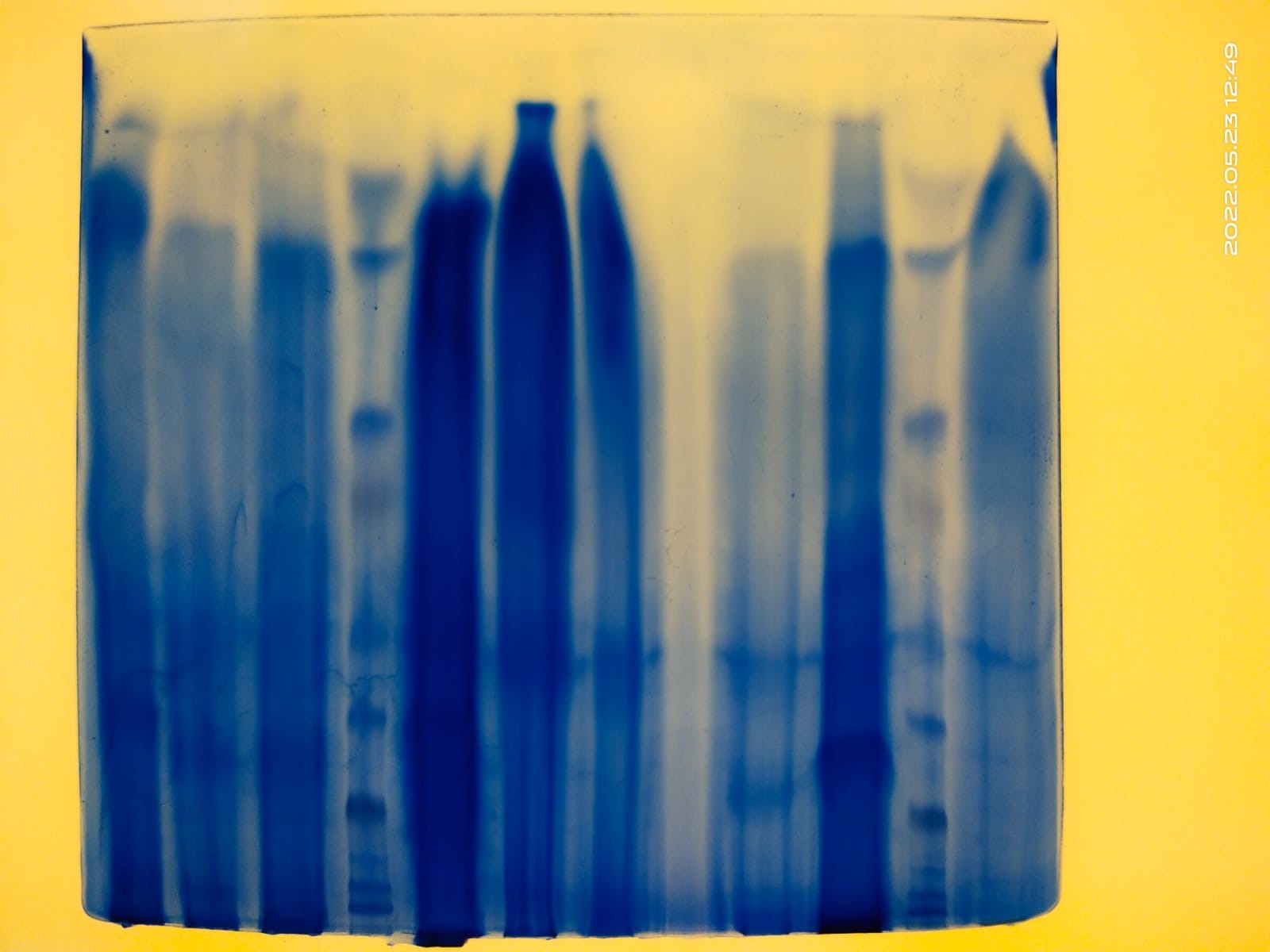


**Figure S3: Full-length gels and blots of the Gel-electrophoresis technique.**

**References**

[1] In. Biotechnology, Broad-Way Prestained Protein Marker Broad-Way Prestained Protein Marker, Cat. No. 2 (n.d.).
